# Supplementary material for: Influenza Vaccination Uptake in the General Italian Population during the 2020–2021 Flu Season: Data from the EPICOVID-19 Online Web-Based Survey
Source: Vaccines (Basel). 2022 Feb 15;10(2):293. doi: 10.3390/vaccines10020293 (PMC8877796; doi:10.3390/vaccines10020293)
Supplement: Supplementary file 1 [file vaccines-10-00293-s001.zip › vaccines-1586546-supplementary.pdf]

Italian regions	Flu shot in 2019/2020	NO	YES	NO	YES	Total
	Flu shot in 2020/2021	NO	NO	YES	YES	
		n (%)	n (%)	n (%)	n (%)	
Abruzzo		211 (63.9)	17 (5.2)	49 (14.8)	53 (16.1)	330 (100)
Basilicata		67 (60.9)	6 (5.5)	12 (10.9)	25 (22.7)	110 (100)
Calabria		114 (57.3)	5 (2.5)	40 (20.1)	40 (20.1)	199 (100)
Campania		500 (56.4)	11 (1.2)	208 (23.5)	167 (18.8)	886 (100)
Emilia-Romagna		2331 (52.0)	171 (3.8)	893 (19.9)	1090 (24.3)	4485 (100)
Friuli-Venezia Giulia		441 (54.3)	37 (4.6)	138 (17.0)	196 (24.1)	812 (100)
Lazio		1958 (48.1)	90 (2.2)	1113 (27.3)	911 (22.4)	4072 (100)
Liguria		737 (49.3)	49 (3.3)	365 (24.4)	343 (23.0)	1494 (100)
Lombardia		7747 (56.0)	680 (4.9)	2584 (18.7)	2821 (20.4)	13832 (100)
Marche		431 (60.7)	17 (2.4)	119 (16.8)	143 (20.1)	710 (100)
Molise		34 (54.0)	1 (1.6)	11 (17.5)	17 (27.0)	63 (100)
Piemonte		2852 (61.4)	174 (3.7)	746 (16.1)	872 (18.8)	4644 (100)
Puglia		468 (51.0)	24 (2.6)	228 (24.8)	198 (21.6)	918 (100)
Sardegna		463 (53.3)	28 (3.2)	188 (21.7)	189 (21.8)	868 (100)
Sicilia		359 (49.7)	16 (2.2)	171 (23.7)	176 (24.4)	722 (100)
Toscana		1667 (52.3)	75 (2.4)	734 (23.0)	712 (22.3)	3188 (100)
Trentino		344 (57.5)	16 (2.7)	127 (21.2)	111 (18.6)	598 (100)
Umbria		188 (60.3)	14 (4.5)	50 (16.0)	60 (19.2)	312 (100)
Valle d'Aosta		50 (65.8)	4 (5.3)	10 (13.2)	12 (15.8)	76 (100)
Veneto		1734 (55.4)	161 (5.1)	549 (17.5)	687 (21.9)	3131 (100)
Other/Unknown		14 (60.9)	0 (0.0)	4 (17.4)	5 (21.7)	23 (100)

Supplementary Table S1. influenza vaccine uptake during the 2019/2020 and 2020/2021 flu seasons according to different Italian regions.

List of abbreviations: n, number.
